# Lack of Resources for Aftercare for Children With Psychosis: A System’s Failure

**DOI:** 10.7759/cureus.12076

**Published:** 2020-12-14

**Authors:** Daisy Shirk, Samantha Horn, Sarah D Williams, Jasmin G Lagman

**Affiliations:** 1 Psychiatry: Child and Adolescent Psychiatry, Penn State Health Milton S. Hershey Medical Center, Hershey, USA; 2 Psychiatry, Penn State Health Milton S. Hershey Medical Center, Hershey, USA; 3 Psychiatry, Philadelphia College of Osteopathic Medicine, Philadelphia, USA

**Keywords:** very early onset schizophrenia (veos), mental health shortage

## Abstract

Very early-onset schizophrenia (VEOS) is a rare disorder that is associated with poor outcomes, especially with securing aftercare plans that will lead to stabilization of illness and prevent recidivism. There is a scarcity of resources available to patients with VEOS and their families once they leave inpatient treatment to achieve long-term success. Here we report a case of a 12-year-old-female who was diagnosed with VEOS at age 11 and since that diagnosis has struggled with finding appropriate resources to meet her needs, requiring frequent hospitalizations and displaying a continued decline in functioning.

## Introduction

Very early-onset schizophrenia (VEOS) is a rare clinical entity that is associated with poor outcomes overall, particularly in securing effective and sustainable care plans for patients suffering from this condition, due to a lack of mental health services and child and adolescent psychiatrists [1]. The World Health Organization reported in 2017 that worldwide, the number of child psychiatrists is less than 0.1 per 100,000 children and adolescents in all income levels except for in high-income countries where the number reaches 1.19 per 100,000 [2]. Skokauskas et al. highlighted the urgency in expanding the workforce of trained professionals, including psychiatrists, clinical psychologists, paediatricians, social workers, nurses, and other healthcare professionals who are all committed to working with children and adolescents with psychiatric illnesses, given the widening gap in current training of these individuals to meet the demands of this unique population [3]. This case emphasizes the reality that the rise in youth mental health conditions has not been met by the expansion of resources and the workforce dedicated to improving outcomes for this population. The American Academy of Child and Adolescent Psychiatry created a workforce map for the United States that breaks down the characteristics of each state and county in terms of several children under 18 years old as well as the number of child and adolescent psychiatrists (CAP) in each area [4]. For Pennsylvania specifically, there are over 2.5 million children under the age of 18 and only 430 CAP, which results in an average of 16 CAP per 100,000 children and adolescents. In the U.S., there are about 15 million children under 18 years who need specialist care from CAP, and there is only about 8,300 CAP in the country.

Kim and The American Academy of Child and Adolescent Psychiatry Task Force on Workforce Need built upon this work to highlight the gaps and barriers to providing the care that children and adolescents with psychiatric disorders need and stressed the fact that the projections about the shortages of child and adolescent psychiatrists are only going to continue to raise concerns and have significant implications in the future [5].

Saxena et al. commented on the widened treatment gap in which many patients with psychiatric conditions do not have access to adequate treatment and resources [6]. They also stressed the socioeconomic differences in access and delivery of psychiatric care across the world, with middle and low-income countries and regions facing the scarcest of resources. However, many higher-income areas are still facing many of the same inequalities. Results from 2016 National Survey on Drug Use and Health demonstrated that only about 4 out of 10 people with a mental health condition had received any kind of treatment over the past year, which can be attributed at least in part to the scarcity of resources and inequities mentioned above [7].

## Case presentation

A 12-year-old female child previously diagnosed with Very Early Onset Schizophrenia (VEOS), who was admitted to the inpatient child psychiatry unit after two prior inpatient admissions following declining functional status and poor medication compliance. She was experiencing worsening verbal and physical aggression towards peers and adults, destruction of property, sexually inappropriate touching, attempting to run into the street into oncoming traffic, and visual and auditory hallucinations. Her symptoms began over the prior year and presented after a months-long decline in overall function, now requiring continuous one-to-one care during the day. Prodromal symptoms included social isolation, declining grades, poor sleep and aggression. Formal IQ testing was performed, which estimated her IQ in the mid-70s. Her family history is significant for schizophrenia in her father, requiring long-term hospitalization for ten years, and her mother also required inpatient psychiatric treatment. Children and Youth Services and Child Protective Services have both been involved with this family due to concerns about neglect and non-compliance with treatment recommendations.

About a month before the patient’s most recent inpatient admission, custody was given to her paternal grandmother for guardianship and medical decision-making. Her grandmother tried to bring her to needed appointments, but it was not enough to keep the patient at home. Psychopharmacological management of this patient consisted of risperidone 1.5mg/day, which was successful in reducing the aggressive behaviours but not the negative symptoms or the impulsive behaviours. Her outpatient care team struggled to find appropriate resources for the patient outside of the inpatient treatment, given the lack of established resources for patients with VEOS. Obtaining services through the Department of Intellectual Disabilities was not possible due to her IQ being above 70. She was referred for a Residential Treatment Facility (RTF) due to her need for constant 1:1 observation for the safety of self and others. However, the team was notified that it was unlikely that she would be accepted into an RTF facility due to her psychosis. Discharging home was a challenge due to the behaviours described above; however, given the lack of resources, this may be the only option available outside of keeping her in the current acute inpatient facility.

## Discussion

VEOS is a rare psychiatric illness with an incidence of less than 0.04% [8]. Patients with VEOS face significant barriers to receiving the care that they require as they get older. Hassan and Taha used a prospective study of children and adolescents with psychosis to assess factors that were related to remission and good outcomes for their patients. They found that acute onset of symptoms, higher IQ, fewer negative symptoms at the onset of the illness, and right medication adherence were all associated with remission and better outcomes for their patients [9]. The need to provide psychosocial supports to patients with VEOS and their families are essential for overall improvement in functioning [8]. One hurdle is that many patients do not achieve a level of functioning to live and work independently, and they often require very long-term treatment. Reichert et al. found that 77.8% of their 27 former patients with childhood schizophrenia were still in outpatient treatment 13.4 years after first admission [10]. Limited resources exist for patients with VEOS to address the complex psychosocial interventions needed to help these patients be successful. Armando et al. described promising preliminary evidence that psychosocial interventions for individuals with very early-onset schizophrenia are an essential component of the comprehensive care that these patients require in order to have the most successful outcomes possible [11]. The review also highlights the paucity of research efforts tailored to the VEOS population that take into account their developmental differences from the early onset and adult-onset schizophrenia. Given the poor prognosis of VEOS, more resources must be devoted to patients and their families to help support them and ensure the best prognosis for these children. 

## Conclusions

There is a global shortage of mental health resources, and this is especially true for children and adolescents. As a result of lack of appropriate resources and delay in obtaining aftercare when it is available, many children and adolescents are having to either remain in acute inpatient hospital settings or are being discharged home with whatever aftercare services are available and with a resulting increase in deterioration upon discharge and increase in recidivism.
